# *SCL20A2* mutation presenting with acute ischemic stroke: a case report

**DOI:** 10.1186/s12883-018-1012-9

**Published:** 2018-01-19

**Authors:** Xiaoyu Zhang, Gaoting Ma, Zhangning Zhao, Meijia Zhu

**Affiliations:** Department of Neurology, Qianfoshan Hospital, Shandong University, Jinan, 250014 China

**Keywords:** Primary familial brain calcification, Stroke, *SLC20A2* mutation

## Abstract

**Background:**

Primary familial brain calcification (PFBC) is a rare disorder characterized by distinctive bilateral brain calcification and variable clinical presentations. However, cerebrovascular attack was rarely reported in PFBC patients. We here reported a *SLC20A2* mutation patient presenting with acute ischemic stroke.

**Case presentation:**

A 56 years old man was transferred to our hospital because of 6 days of melena and 3 days of somnolence, agitation and mood changes. Computed tomography (CT) scan showed symmetrical calcifications in bilateral basal ganglia, caudate nucleus, thalami, subcortical white matter and cerebellum, which is consistent with PFBC. Brain magnetic resonance imaging (MRI) revealed acute ischemic stroke in bilateral basal ganglia and periventricular regions. Mutational analysis identified a *SLC20A2* gene mutation c.344C > T (p.Thr115Met) in exon 3. One of his daughters had also suffered from brain calcification. MR perfusion imaging revealed hypoperfusion in bilateral basal ganglia, prefrontal and temporal lobe. After treatment, he discharged with a favorable functional outcome but cognitive impairment.

**Conclusions:**

Ischemic stroke can occur in PFBC patients, which may be associated with hypoperfusion and calcification of arteries. And hypoperfusion in frontotemporal lobar may be related with their cognitive impairment.

## Background

Primary Familial Brain Calcification (PFBC), also known as Fahr’s disease (FD), is a rare disorder characterized by symmetrical calcification in the basal ganglia and other brain regions. The disorder was first described by Delacour in 1850 and the best-known case description was by Fahr in 1930 [1, 2]. Patients with PFBC mostly present with movement disorders, psychiatric symptoms or cognitive impairment. Although the exact mechanisms of PFBC remain unclear, recent discoveries of four causative genes namely *SCL20A2, PDGFB, PDGFRB*, and *XPR1* may offer important clues [3–6]. *SLC20A2* gene encodes for type III sodium-dependent phosphate transporter 2 (PiT2). *PDGFB* and *PDGFRB* genes are related with function of pericytes and integrity of blood brain barrier (BBB) [7]. It has been postulated that disturbance of neuronal calcium phosphorus metabolism and disruption of BBB in PFBC patients may contribute to metastatic deposition and cause intracranial calcification [8].

In pathological studies, calcium and other mineral deposits have been found in the walls of capillaries, arterioles and the perivascular spaces in patients with PFBC. Furthermore, small number of arteries may have severe calcification which could cause obstruction of the lumen [9]. It can be assumed that those brain regions with affected arteries may be easily attacked by ischemic stroke. However, ischemic stroke was rarely reported in PFBC patients. The relationship between PFBC and cerebrovascular disease is still confusing. Therefore, we here report a 55-year-old male with *SCL20A2* mutation presenting with acute ischemic stroke, and we further explore the relationship between PFBC and ischemic stroke.

## Case presentation

**Fig. 1:**
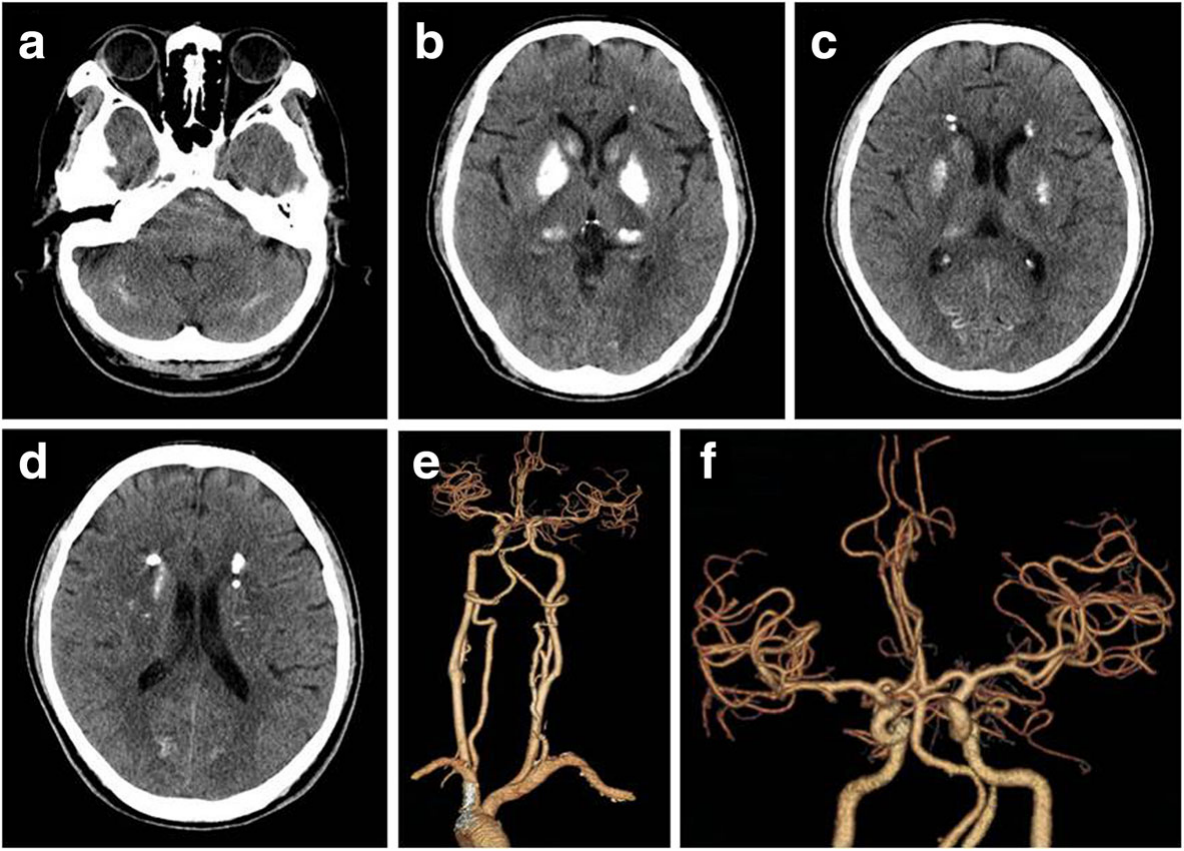
Computed tomography (CT) and CT angiography (CTA). **a**-**d** symmetric calcifications in bilateral basal ganglia, caudate nucleus, thalami, subcortical white matter and cerebellum; **e**-**f** arteriosclerosis of cervical and cerebral arteries

**Fig. 2:**
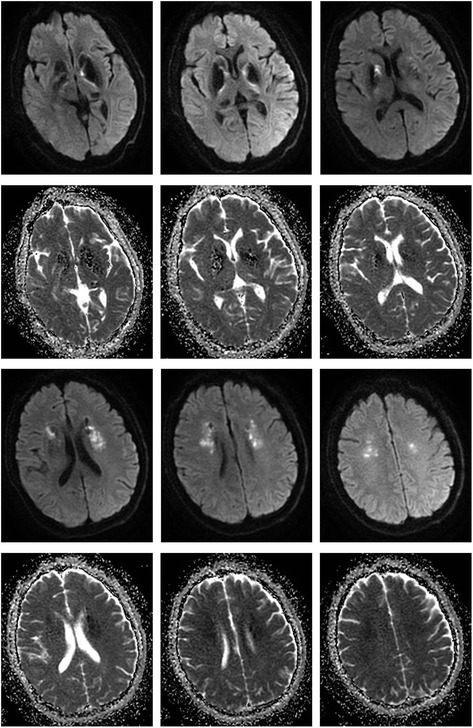
Brain magnetic resonance imaging (MRI). Hyperintense signal in diffusion weighted imaging (DWI) and hypointense signal in apparent diffusion coefficient (ADC) in bilateral basal ganglia and periventricular regions

A 56 years old man, with histories of lacunar stroke and gastric ulcer, came to our hospital because of 6 days of melena and 3 days of somnolence, agitation and mood changes. The patient was a farmer and did not take any antiplatelet and antacids drugs. He came to local community hospital for dizzy and melena on June 24th, 2017. Blood examination showed anemia with 73 g/L of hemoglobin. He was treated with proton pump inhibitors. Two days later, he recovered from melena but presented with somnolence. Blood test was reexamined and showed aggravation of anemia with 58 g/L of hemoglobin. He was treated with red cell transfusions and then transferred to our hospital. On admission, vital signs were: blood pressure 96/48 mmHg, pulse 48 beats/min, respirations 12 breaths/min, body temperature 36.8 °C. The neurologic examination revealed somnolence, aphasia, bilateral decreased muscle power in grade 4 and bilateral babinski sign. He presented with agitation when he was awaken. The score of National Institutes of Health Stroke Scale (NIHSS) was 11 points. Computed tomography (CT) scan showed symmetrical calcifications in bilateral basal ganglia, caudate nucleus, thalami, subcortical white matter and cerebellum (Fig. 1). Brain magnetic resonance imaging (MRI) revealed acute ischemic stroke in bilateral basal ganglia and periventricular regions with hyperintense signal in diffusion weighted imaging (DWI) and hypointense signal in apparent diffusion coefficient (ADC) (Fig. 2). The patient was admitted to neurological department with the diagnosis of acute ischemic stroke.

**Fig. 3:**
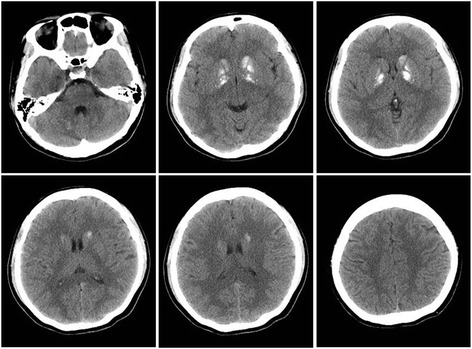
Computed tomography (CT) of a daughter with brain calcification. Symmetric calcifications in bilateral basal ganglia, caudate nucleus, subcortical white matter and cerebellum

At hospitalization, possible causes of intracranial calcification were investigated. The level of serum calcium was 2.08 mmol/L (normal range: 2.10–2.55 mmol/L). The level of serum phosphorus, copper, ceruloplasmin, thyroid hormone, parathyroid hormone and enzyme-linked immunosorbant assay for human immunodeficiency virus (HIV) were normal. Family history revealed that one of his three daughters (34 years old) had also suffered from brain calcification, but without neurological problems (Fig. 3). His parents and two brothers also have no neurological problems. His father died of cardiac disease 10 years ago and his mother died of cancer 13 years ago. Mutational analysis was performed with a sample of whole blood from the patient and showed a *SLC20A2* gene mutation c.344C > T (p.Thr115Met) in exon 3. Therefore, a diagnosis of PFBC was made.

**Fig. 4:**
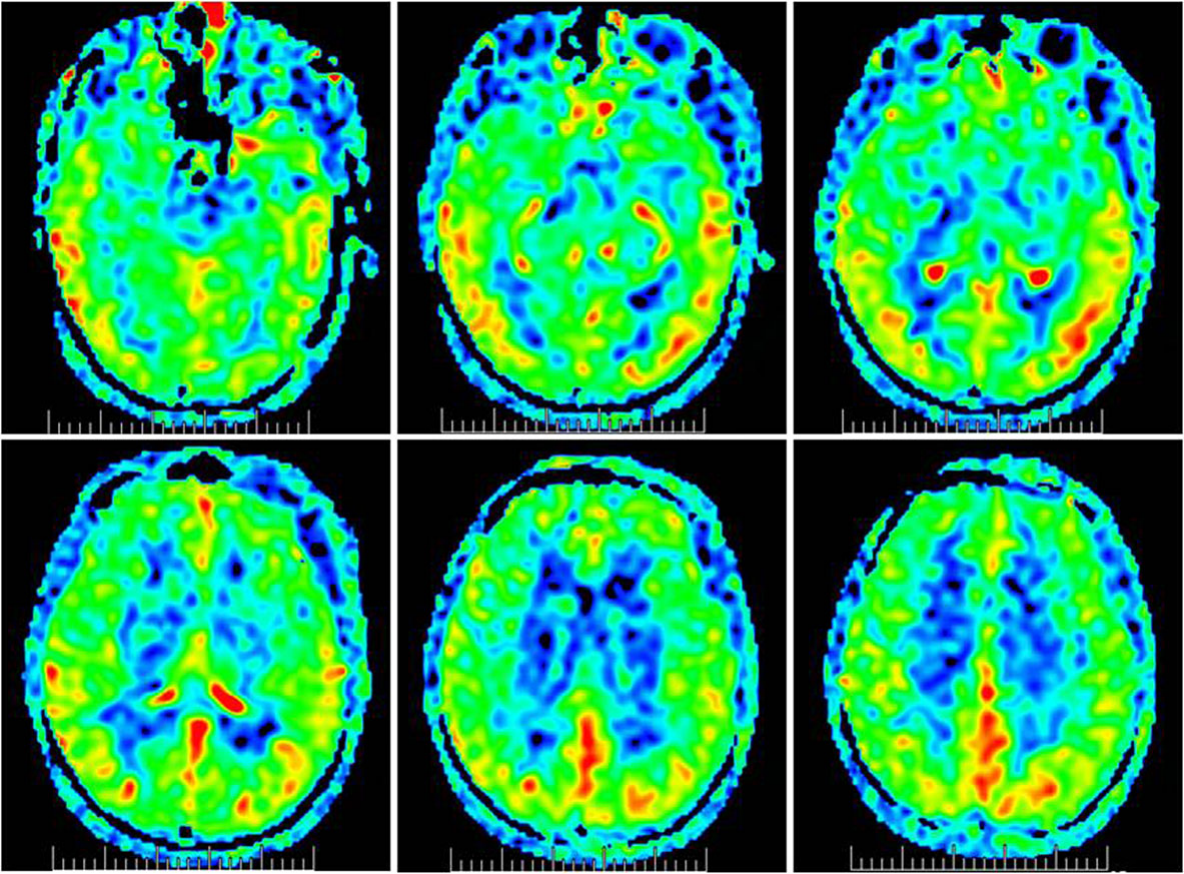
MR perfusion imaging. Hypoperfusion in bilateral basal ganglia, prefrontal and temporal lobe

In order to identify the possible mechanism of ischemic stroke, CT angiography of cervical and cerebral arteries were performed and showed arteriosclerosis (Fig. 1). Cerebral microbleeds were not observed in susceptibility weighted imaging (SWI) and cerebral veins were normal on magnetic resonance venography (MRV). Sinus rhythm was showed on electrocardiogram. Mural thrombus was not detected in ultrasound cardiogram. We hypothesized that ischemic stroke may be related with hypoperfusion. The level of low density lipoprotein was 2.78 mmol/L at admission. Risuvastatins were given according to the guideline of acute ischemic stroke. Antacids and volume expansion therapy were regularly administrated. Five days after admission, the patient presented with a normal state of consciousness and could speak simple words. However, his memory was not completely recovered. MR perfusion imaging was taken and revealed that there was hypoperfusion in bilateral basal ganglia, prefrontal and temporal lobe (Fig. 4). After treatment for 20 days, he discharged with a favorable functional outcome and could carry out daily activities independently. The score of NIHSS was one point (correctly answered one question when he was asked his age and the name of the current month) and modified Rankin Scale score was two points (unable to carry out all pre-stroke activities, such as working as a farmer). However, he still had cognitive impairment with the score of 18 by mini-mental state examination (MMSE). The level of hemoglobin was 90 g/L at discharge.

## Discussion and conclusions

Primary familial brain calcification, or Fahr’s disease, is a rare disorder characterized by distinctive bilateral brain calcification and variable clinical presentations. Four genes have been known to be associated with PFBC, including *SCL20A2, PDGFB, PDGFRB* and *XPR1*. *SLC20A2* is the most common gene involved with PFBC (55–66%), followed by *PDGFB* (31%) and *PDGFRB* (10%) [10]. It has also been reported that 14% patients with PFBC were sporadic cases. To date, about 40 variants in *SLC20A2* gene have been discovered [8]. In our patient, the mutation of c.344C > T (p.Thr115Met) in exon 3 has also been reported before [11].

The clinical presentations in PFBC patients are variable and almost a quarter of cases are asymptomatic. In previous studies, the most frequently described motor signs in PFBC patients were parkinsonism and dystonia [12]. Moreover, dysarthria, ataxia and chorea had also been described in published literatures [10]. Psychiatric features in PFBC patients include cognitive impairment, mood disorders, psychotic symptoms and obsessive-compulsive symptoms. In patients with *SLC20A2* mutations, the mostly described clinical presentations were parkinsonism, dystonia and cognitive impairment [12]. In our patient, parkinsonism was not observed. However, he may have cognitive impairment before stroke because of forgetfulness provided by his daughters. And no clinical symptoms were observed in his daughter with brain calcification. In clinical practice, other causes of intracranial calcification should be distinguished from PFBC, including physiological calcification, disorders of calcium metabolism (hypoparathyroidism, pseudo-hypoparathyroidism, pseudo-pseudo-hypoparathyroidism and hyperparathyroidism), mitochondrial diseases, severe hypomagnesemia, HIV infection and other toxic conditions. Detailed medical histories and laboratory tests are helpful in determining the causes of intracranial calcification.

Cerebrovascular attack was rarely reported in PFBC patients. As far as we know, only two cases were reported in recent literatures, one patient with subarachnoid hemorrhage and the other with lacunar stroke in left genu of the internal capsule [13, 14]. The underlying mechanisms of ischemic stroke in PFBC patients are still unclear. In pathologic studies, calcium and other mineral deposits were found in the walls of capillaries, arterioles and in the perivascular spaces [9, 15]. A small number of these arteries even exhibited obstruction of the lumen because of severe calcification. Reactive astrocytes and microglia accumulated around the calcified deposits, indicating a mild ongoing inflammatory process. Furthermore, microangiopathy was observed in skin biopsy of a patient with *PDGFB* mutation, showing thickened and fragmented areas in the basal lamina [16]. These changes may contribute to vascular stenosis and cause brain hypoperfusion. Evidences from neuroimagings also supported this assumption. Hypoperfusion were detected in basal ganglia region by single photon emission computed tomography (SPECT) and massive reduction of glucose metabolism were observed by position emission tomography (PET) [17, 18]. Therefore, it may be reasonable to postulate that vascular stenosis develop in affected vessels because of extensive calcium and mineral deposits, and ischemic stroke can occur once cerebral perfusion decreased. In our case, hypovolemia caused by gastrointestinal bleeding may result in hypoperfusion of brain and cause acute ischemic stroke in bilateral basal ganglia and periventricular regions. We also excluded other etiologies which may cause bilateral multiple ischemic stroke such as embolism, cerebral amyloid angiopathy and immunologically-mediated small vessel diseases.

Cognitive impairment is often observed in PFBC patients, while the underlying mechanisms are still largely unknown. In MR morphological evaluation, atrophy was detected in the left orbitofrontal gyrus and left hippocampus, which may affect their cognitive function [19]. In other studies, reduced perfusion by SPECT and decreased glucose metabolism by PET were found in frontal and temporal brain region, which is consistent with our MR perfusion results [17, 18]. Moreover, PFBC patients may presented with decreased resting state functional connectivity in executive control and working memory networks by functional MRI (fMRI) [19]. Therefore, both structural and functional network changes associated with frontotemporal lobar may contribute to cognitive impairment in PFBC patients. In our case, the patient presented with moderate dementia at discharge which may be related with decreased perfusion in frontotemporal lobar. However, it should be noted that many PFBC patients have no cognitive impairment, indicating that other factors may involve in the processes. More detailed studies are needed in the future.

In summary, we here presented acute ischemic stroke in a PFBC patient with *SLC20A2* gene mutation. The possible mechanism of ischemic stroke in PFBC patient may be associated with hypoperfusion and calcification of arteries. Furthermore, PFBC patient can present with cognitive impairment which may be attributed to structural and functional network changes of frontotemporal lobar.

## Abbreviations

ADC: Apparent diffusion coefficient
BBB: Blood brain barrier
CT: Computed tomography
DWI: Diffusion weighted imaging
FD: Fahr’s disease
FLAIR: Fluid attenuated inversion recovery
fMRI: functional MRI
HIV: Human immunodeficiency virus
MMSE: Mini-mental state examination
MRI: Magnetic resonance imaging
NIHSS: National Institutes of Health Stroke Scale
PET: Position emission tomography
PFBC: Primary familial brain calcification
PiT2: Sodium-dependent phosphate transporter 2
SPECT: Single photon emission computed tomography
SWI: Susceptibility weighted imaging
